# Tumour-derived alkaline phosphatase regulates tumour growth, epithelial plasticity and disease-free survival in metastatic prostate cancer

**DOI:** 10.1038/bjc.2016.402

**Published:** 2016-12-22

**Authors:** S R Rao, A E Snaith, D Marino, X Cheng, S T Lwin, I R Orriss, F C Hamdy, C M Edwards

**Affiliations:** 1 Nuffield Department of Surgical Sciences, University of Oxford, Oxford OX3 7LD, UK; 2 Nuffield Department of Orthopaedics, Rheumatology and Musculoskeletal Sciences, Botnar Research Centre, University of Oxford, Oxford OX3 7LD, UK; 3 Royal Veterinary College, University of London, London NW1 0TU, UK

**Keywords:** prostate cancer, metastasis, alkaline phosphatase, bone, EMT, MET

## Abstract

**Background:**

Recent evidence suggests that bone-related parameters are the main prognostic factors for overall survival in advanced prostate cancer (PCa), with elevated circulating levels of alkaline phosphatase (ALP) thought to reflect the dysregulated bone formation accompanying distant metastases. We have identified that PCa cells express *ALPL*, the gene that encodes for tissue nonspecific ALP, and hypothesised that tumour-derived *ALPL* may contribute to disease progression.

**Methods:**

Functional effects of *ALPL* inhibition were investigated in metastatic PCa cell lines. *ALPL* gene expression was analysed from published PCa data sets, and correlated with disease-free survival and metastasis.

**Results:**

*ALPL* expression was increased in PCa cells from metastatic sites. A reduction in tumour-derived *ALPL* expression or ALP activity increased cell death, mesenchymal-to-epithelial transition and reduced migration. Alkaline phosphatase activity was decreased by the EMT repressor Snail. In men with PCa, tumour-derived *ALPL* correlated with EMT markers, and high *ALPL* expression was associated with a significant reduction in disease-free survival.

**Conclusions:**

Our studies reveal the function of tumour-derived *ALPL* in regulating cell death and epithelial plasticity, and demonstrate a strong association between *ALPL* expression in PCa cells and metastasis or disease-free survival, thus identifying tumour-derived *ALPL* as a major contributor to the pathogenesis of PCa progression.

Prostate cancer is the second most common cancer in men, with an estimated 1.1 million men diagnosed with prostate cancer in 2012 worldwide (Torre *et al*, 2015). Whereas localised prostate cancer has a good prognosis, advanced disease metastasises frequently to the skeleton, and becomes castration resistant. Once prostate cancer has metastasised to distant sites, the disease is ultimately fatal and treatment is purely palliative. The most common metastatic sites are the lymph nodes and the bone, with bone metastases resulting in severe bone pain and fractures (Gartrell and Saad, 2014). Despite many advances in research, a greater understanding of the metastatic process is required, to identify new therapeutic targets.

Recent evidence suggests that bone-related parameters are the main prognostic factors for overall survival in advanced prostate cancer. These include alkaline phosphatase, bone-specific alkaline phosphatase, urinary *n*-telopepetide, previous skeletal-related events and pain (Metwalli *et al*, 2014; Fizazi *et al*, 2015; Koo *et al*, 2015). Alkaline phosphatase, also known as tissue nonspecific alkaline phosphatase (TNAP) or mesenchymal stem cell antigen 1, is an enzyme that is expressed by a range of tissues including bone, and also the liver (Buchet *et al*, 2013). There are four existing isoforms of alkaline phosphatase, and the *ALPL* gene encodes for TNAP. Alkaline phosphatase is highly expressed by osteoblasts and a key component of osteoblastic activity, acting to hydrolyse inorganic pyrophosphate resulting in mineralisation (Millan, 2013). The bone lesions associated with prostate cancer are predominantly osteosclerotic, arising because of an increase in osteoblastic activity and uncontrolled formation of new bone. As such, the elevated circulating levels of alkaline phosphatase seen in advanced prostate cancer are thought to reflect the dysregulated bone formation associated with cancer-induced bone disease.

Advanced prostate cancer is associated with the necessity to escape from the primary site, typically associated with increased cellular migration and epithelial-to-mesenchymal transition (EMT). Epithelial-to-mesenchymal transition is characterised by a change in cellular morphology, a loss of epithelial markers such as E-cadherin and a corresponding increase in mesenchymal markers. Although the dependence of metastasis upon EMT has recently been questioned in lung and pancreatic cancer, epithelial plasticity remains a fundamental trait of metastatic tumour cells (Brabletz, 2012; Fischer *et al*, 2015; Zheng *et al*, 2015). Upon successful colonisation of the bone, there is evidence of a mixed epithelial–mesenchymal phenotype, highlighting the importance of both EMT and mesenchymal-to-epithelial transition (MET) in metastasis (van der Pluijm, 2011). Within the tumour-bone microenvironment, it is known that cancer cells can express markers typically associated with bone cells, including osteopontin, osteocalcin, RUNX2 and RANKL (Koeneman *et al*, 1999; Brubaker *et al*, 2003; Zayzafoon *et al*, 2004; Huang *et al*, 2005; Fradet *et al*, 2013). Expression of bone-related markers, such as RUNX2, has been shown to promote the bone-metastatic phenotype of prostate cancer cells (Baniwal *et al*, 2010). However, in contrast to RUNX2, the expression, regulation and function of the defining osteoblastic enzyme alkaline phosphatase in tumour cells is poorly understood. In this study, we have identified *ALPL* expression in metastatic prostate cancer cells, with high *ALPL* expression associated with a reduction in disease-free survival in patients with prostate cancer. We have undertaken studies to elucidate the function of tumour-derived alkaline phosphatase in the biology of prostate cancer, and identified a novel role in both cell death and epithelial plasticity.

## Materials and Methods

### Cell lines and reagents

ARCaP, ARCaPE and ARCaPM human prostate cancer cells lines were purchased from Novicure Inc. (Birmingham, AL, USA). All three cell lines were routinely maintained in MCaP medium (Novicure; cat. no. 3300) with 5% foetal bovine serum (FBS) and penicillin–streptomycin, unless otherwise indicated. C4-2B human prostate cancer cells were a kind gift from Prof. George Thalmann (University of Bern, Switzerland), and maintained in RPMI-1640 medium with 10% FBS, vitamins, non-essential amino acids, L-glutamine, sodium pyruvate and penicillin–streptomycin (complete RPMI medium), unless otherwise indicated.

PC3 human prostate cancer cell line, obtained from American Type Culture Collection (ATCC, Teddington, UK), were routinely maintained in complete RPMI medium (Sigma-Aldrich, Gillingham, UK) containing 10% FBS and penicillin–streptomycin. All other prostate cancer cell lines were a kind gift from Dr Richard Bryant (University of Oxford, Oxford, UK). 2T3 mouse preosteoblast cells were obtained from ATCC and maintained in RPMI-1640 medium with 10% FBS and antibiotics, unless otherwise indicated. Where possible, cell lines were validated by genotyping. Periodic testing ensured the absence of mycoplasma contamination in all cell lines. Apyrase (A6535) and porcine kidney alkaline phosphatase (P4439) were obtained from Sigma (Gillingham, UK).

### Plasmid DNA transfection

Plasmid DNA was transfected using Lipofectamine 2000 (Life Technologies, Renflew, UK) according to the manufacturer's protocol. Typically, 24 *μ*g of plasmid DNA in 1 ml of Opti-MEM (Life Technologies) was mixed with 60 *μ*l of Lipofectamine 2000 in 1 ml of Opti-MEM and incubated for 5–10 min at room temperature. The DNA-Lipofectamine complexes were then added to cells at 80–90% confluence in 10cm dishes in 10 ml of antibiotic-free growth medium.

### Small interfering RNA transfection

Small interfering RNA were transfected in prostate cancer cells using Lipofectamine RNAiMax (Life Technologies) according to the manufacturer's protocol. Details of siRNAs are as follows: non-targeting control pool, cat. no. D-001206-13-05; *ALPL*-targeting ON-TARGETplus siRNA pool, cat. no. L-008658-00-0005; SNAI1-targeting ON-TARGETplus siRNA pool, cat. no. L-010847-01-0005.

### Lentiviral packaging and transduction

Lentiviral packaging was performed using 293T cells using a third-generation packaging system. Briefly, 10 *μ*g of transfer vector, 5 *μ*g of pMDLg/pRRE, 2.5 *μ*g of pRSV-Rev and 2.5 *μ*g of pCMV-VSVG were transfected into 293T cells using Lipofectamine 2000 in a 10 cm tissue culture dish, and 3 days later, the culture supernatant containing the lentivirus was collected. The lentivirus was made into a 10 × concentrated stock using the Lenti-X Concentrator Solution (Clontech, Mountain View, CA, USA; 631232) and was used at a dilution of 1 : 10 in the cell suspension for transduction. Hexadimethrine bromide (Sigma) was added at a concentration of 10 *μ*g ml^−1^ in the cell suspension, to enhance transduction efficiency. The transfer vectors are scrambled control (Sigma) or ALPL targeting short hairpin RNAs (Sigma; cat. no. SHCLNG-NM 000478; TRCN0000052003: 5′-CCGGCCCACAATGTGGACTACCTATCTCGAGATAGGTAGTCCACATTGTGGGTTTTTG-3′, TRCN0000052004: 5′-CCGGCGTGGCTAAGAATGTCATCATCTCGAGATGATGACATTCTTAGCCACGTTTTTG-3′, TRCN0000052005: 5′-CCGGGCGCAAGAGACACTGAAATATCTCGAGATATTTCAGTGTCTCTTGCGCTTTTTG-3′, TRCN0000359258: 5′-CCGGATGTCTCCATGGTGGACTATGCTCGAGCATAGTCCACCATGGAGACATTTTTTG-3′, TRCN0000359332: 5′-CCGGAGTATGAGAGTGACGAGAAAGCTCGAGCTTTCTCGTCACTCTCATACTTTTTTG-3′), designated as shSCR and shALPL1-shALPL5, respectively. These plasmids have a pLKO.1 backbone, which is suitable for both second- and third-generation packaging systems.

### RNA isolation

Total RNA was isolated from cultured cells using Trizol reagent (Life Technologies; 15596026), according to the manufacturer's protocol. Quality of the RNA was assessed by measuring the ratios of absorbance (*A*_260_/*A*_280_ and *A*_260_/*A*_230_); ratios >1.8 were considered suitable for further analysis.

### Real-time qRT–PCR

For quantitative reverse transcription–polymerase chain reaction (qRT–PCR) of mRNA, 1 *μ*g of DNAse I-treated RNA was reverse transcribed using iScript cDNA Synthesis Kit (Bio-Rad, Hemel Hempstead, UK; 1708890). This cDNA was appropriately diluted and was used in a qPCR reaction with Fast SYBR Green Real-time Mastermix (Life Technologies; 4385612). ALPL: F, 5′-ACGTGGCTAAGAATGTCATC-3′ and R, 5′-CTGGTAGGCGATGTCCTTA-3′ CDH1: F, 5′-AGGCCAAAGCAGCAGTACATT-3′ and R, 5′-ATTCACATCCAGCACATCCA-3′ GAPDH QuantiTect Primer Assay No. QT01192646 (Qiagen); KRT14: F, 5′-GGCCTGCTGAGATCAAAGACT-3′ and R, 5′-TCTGCAGAAGGACATTGGCAT-3′ SNAI1: QuantiTect Primer Assay No. QT00010010 (Qiagen); VIM: F, 5′-CCTTGAACGCAAAGTGGAATC-3′ and R, 5′-GACATGCTGTTCCTGAATCTGAG-3′ ZEB1: F, 5′-CCAGGGAGGAGCAGTGAAAG-3′ and R, 5′-CCCCAGGATTTCTTGCCCTT-3′.

### Protein isolation and western blotting

Cultured cells were lysed with CelLytic M Cell Lysis Reagent (Sigma; C2978) containing a protease inhibitor cocktail (Sigma; S8830). Protein concentration was quantified using a Pierce Bicinchonic Acid Assay Kit (Life Technologies). Western blotting was performed as described previously, using E-cadherin, vimentin and ZEB1 antibodies from the EMT Antibody Sampler Kit (Cell Signaling; 9782) and *β*-actin antibody (Sigma; A5316).

### Cell viability

Viability of cultured cells *in vitro* was determined using the Alamar Blue assay. Briefly, a working solution (1 mg ml^−1^) of resazurin (Sigma; R7017) was added to the cultured cells at a ratio of 1 : 10 with respect to the volume of growth medium (10 *μ*l in each well of a 96-well plate containing 100 *μ*l of growth medium). The cells were then incubated in a humidified CO_2_ incubator at 37 °C until a change in colour was visibly noticeable (or for a duration of 2.5 h for time-course experiments) and the plate was read using a fluorimeter (FLUOstar, BMG Labtech, Aylesbury, UK) (excitation: 545 nm and emission: 585 nm).

### YO-PRO-1 staining

YO-PRO-1 iodide stain (Life Technologies; cat. no. Y3603) (stock concentration: 1 mM in DMSO) was added to the growth medium of cells growing in 96-well plates, to a final concentration of 1 *μ*M (1 : 1000 dilution). The cells were then incubated in a humidified CO_2_ incubator at 37 °C for 60 min and the plate was read using a fluorimeter (excitation: 490 nm and emission: 510 nm). Live cells do not take up YO-PRO-1; therefore, changes in expression are indicative of cell death.

### Flow cytometry

Transfected cells were washed with phosphate-buffered saline and resuspended in Annexin Binding Buffer (ThermoFisher Scientific; V13246) at a concentration of 10^6^ cells per ml. The cells were then stained with Annexin V-Pacific Blue (ThermoFisher Scientific; A35122) and propidium iodide (PI) according to the manufacturer's specifications and incubated at room temperature for 15 min, following which they were analysed using a BD Fortessa flow cytometer (BD Biosciences, Oxford, UK). Early apoptotic cells were defined as positive for Annexin V, but negative for PI.

### Staining for alkaline phosphatase activity

Staining for alkaline phosphatase activity was performed using the Leukocyte Alkaline Phosphatase Kit (Sigma; 86C) according to the manufacturer's protocol.

### Quantitative measurement of alkaline phosphatase activity

Cell lysates were obtained as described above. A 20 × stock solution was made by dissolving 1.025 mg ml^−1^ of 4-methylumbelliferyl phosphate (substrate for alkaline phosphatase; Sigma; cat. no. M8168) in 50 mM Tris (pH 8) buffer. A working solution was made by diluting the stock solution with 50 mM Tris (pH 8) buffer. To 60 *μ*l cell lysate or conditioned medium in an opaque 96-well plate, 100 *μ*l of working substrate solution was added and incubated in the dark at 37 °C for 45 min. Subsequently, the reaction was stopped by adding 100 *μ*l of 1 M Na_2_CO_3_ and the plate was read in a fluorimeter at an excitation wavelength of 360 nm and an emission wavelength of 450 nm.

### Statistics

All statistical analyses for *in vitro* experiments were performed using GraphPad Prism (Graphpad Software Inc., La Jolla, CA, USA). Comparisons between two groups of data were performed using two-tailed unpaired Student's *t*-test. Variances of data between the two groups were compared using an F-test; where there was unequal variance between the two groups, Welch's correction was used. For comparisons between more than two groups, multiple t test with Holm–Sidak correction was used. In all cases, significance was considered at *α*<0.05. Analysis of previously published prostate cancer data sets was performed in R. The Taylor *et al.* data set (Taylor *et al*, 2010) was downloaded from cBioportal (http://www.cbioportal.org/) and the Tomlins *et al.* data set (Tomlins *et al*, 2007) was downloaded from Gene Expression Omnibus (accession no.: GDS3289). For survival analysis, samples were stratified into ‘high' and ‘low' expressers using the median expression for the respective genes as the cutoffs.

## Results

### Alkaline phosphatase is expressed by prostate cancer cells and increased in metastatic prostate cancer

**Figure 1:**
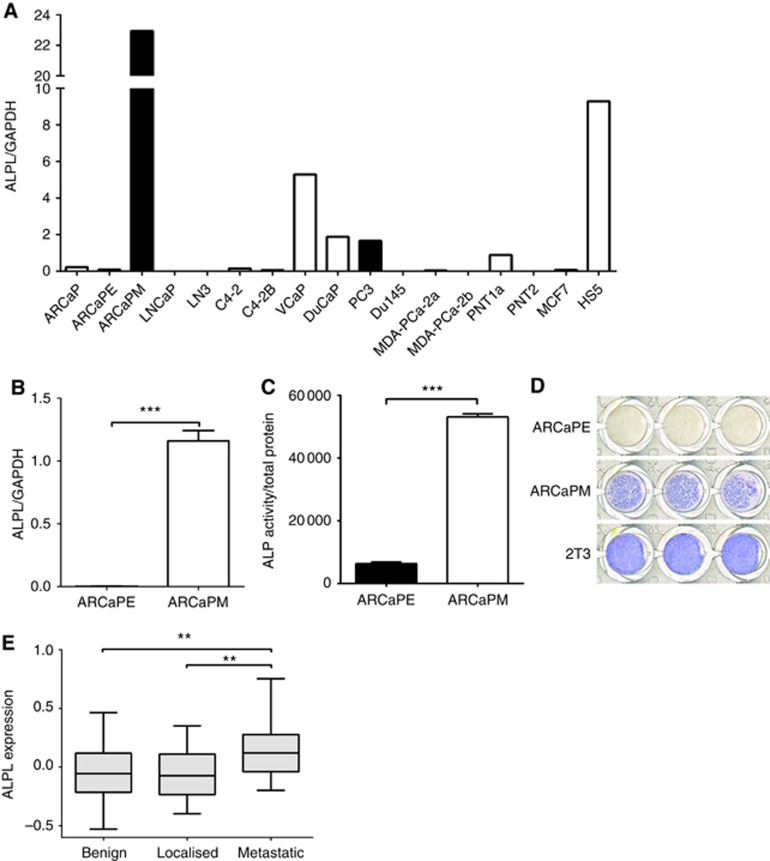
**Alkaline phosphatase is expressed by prostate cancer cells and increased in metastatic prostate cancer.** (**A**) *ALPL* mRNA expression (normalised with GAPDH (glyceraldehyde 3-phosphate dehydrogenase) expression) was measured using qRT–PCR in a panel of prostate cancer cell lines, the breast cancer cell line MCF7 and the bone marrow stromal cell line HS5. Bars for metastatic cell lines (ARCaPM, C4-2B and PC3) are shaded in black. (**B**) *ALPL* mRNA expression (normalised with GAPDH expression) was measured using qRT–PCR and (**C**) alkaline phosphatase activity (normalised with total protein) was measured using a quantitative fluorimetric assay, in ARCaPE and ARCaPM cells. (**D**) ARCaPE, ARCaPM and 2T3 cells were stained for alkaline phosphatase activity. (**E**) The Tomlins *et al.* data set (Tomlins *et al*, 2007) was analysed for *ALPL* mRNA expression in benign prostatic hyperplasia, localised prostate cancer and metastatic prostate cancer samples. (**P<0.01, ***P<0.001).

Circulating alkaline phosphatase is well known to be elevated in prostate cancer-induced bone disease, indicative of osteoblast-derived alkaline phosphatase resulting in increased bone formation. In contrast, the expression and function of tumour-derived alkaline phosphatase is poorly understood. To determine the expression of alkaline phosphatase specifically in prostate cancer cells, we first examined a panel of prostate cancer cell lines that vary in their *in vivo* metastatic potential, demonstrating high gene expression in some, but not all, metastatic prostate cancer cell lines (Figure 1A). Low to non-existent expression was observed in the non-tumourigenic normal human prostate epithelium cell lines PNT1A and PNT2, and in MCF-7 breast cancer cells, previously demonstrated to exhibit low basal levels of alkaline phosphatase expression (Tsai *et al*, 2000). Using the paired cell lines ARCaPE and ARCaPM, which differ in their metastatic ability, we demonstrated a significant increase in *ALPL* expression and alkaline phosphatase enzymatic activity in metastatic ARCaPM cells as compared with non-metastatic ARCaPE cells, with prostate cancer cells demonstrating increased alkaline phosphatase activity per mg protein as compared with 2T3 preosteoblasts (Figure 1B–D, Supplementary Data Figure S1). We also observed differences in the ability of ARCaPE and ARCaPM cells to mineralise, with only ARCAPM cells staining positively for Alizarin Red, indicating the presence of calcium deposits and mineralisation (Supplementary Data Figure S2). To determine the clinical relevance of alkaline phosphatase expression in prostate cancer cells, we analysed gene expression data from a publicly available data set in which prostate cancer cells from primary and metastatic sites were isolated by laser capture microdissection (Tomlins *et al*, 2007). A significant increase in *ALPL* expression was detected in metastatic prostate cancer cells as compared with localised prostate cancer (Figure 1E).

### Inhibition of alkaline phosphatase activity reduces prostate cancer cell viability and induces apoptosis

**Figure 2:**
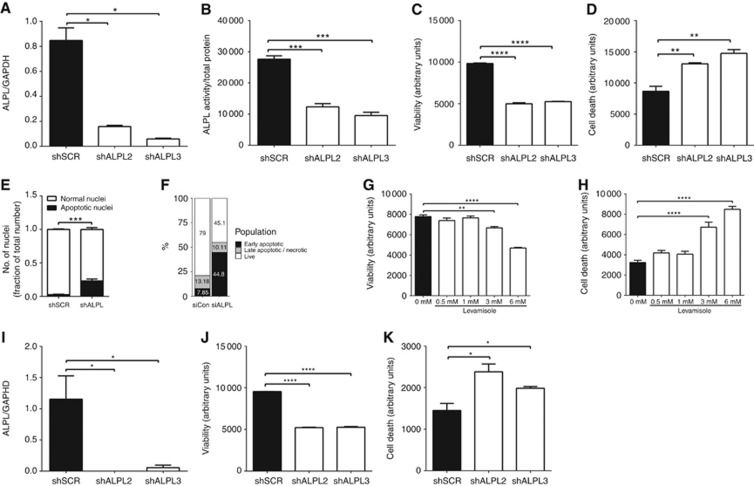
**Inhibition of alkaline phosphatase activity decreases cell viability and increases apoptosis.** Knockdown of alkaline phosphatase was confirmed in ARCaPM cells transduced with scrambled shRNA (shSCR) or *ALPL*-knockdown shRNA (shALPL2 and shALPL3) by measuring (**A**) *ALPL* mRNA using qRT–PCR and (**B**) alkaline phosphatase activity (normalised with total protein) using a quantitative fluorimetric assay, 72 h after transduction. (**C**) Viability of the transduced cells was measured using Alamar Blue assay and (**D**) cell death was measured using YO-PRO-1. (**E**) Apoptotic nuclei, identified from their fragmented appearance, were counted in ImageJ (imagej.nih.gov). (**F**) Apoptosis was quantitated using annexin V/PI staining. Viability (**G**) and (**H**) cell death were also measured in ARCaPM cells treated with increasing concentrations of levamisole using Alamar Blue assay and YO-PRO-1 staining, respectively. (**I**) ALPL knockdown in PC3 cells was confirmed using qRT–PCR and (**J**) the effects of viability were measured using Alamar Blue assay and (**K**) cell death was measured using YO-PRO-1 staining. (*P<0.05, **P<0.01, ***P<0.001, ****P<0.0001).

To begin to determine the functional role of alkaline phosphatase in prostate cancer biology, we took molecular and pharmacological approaches to inhibit *ALPL* gene expression and alkaline phosphatase enzymatic activity. Following lentiviral delivery of *ALPL* shRNA (or scrambled control) to ARCaPM cells, we confirmed a significant decrease in alkaline phosphatase gene expression and enzymatic activity (Figures 2A and B). Knockdown of *ALPL* resulted in a significant reduction in cell viability (Figure 2C and D and Supplementary Data Figure S3) and an increase in apoptotic cell death (Figure 2E and F, Supplementary Data Figure S4). *ALPL* knockdown cells did not survive for long-term culture (data not shown). The pharmacological inhibitor of alkaline phosphatase activity levamisole was shown to inhibit alkaline phosphatase activity *in vitro* (Supplementary Data Figure S5), and treatment of ARCaPM cells with levamisole resulted in a dose-dependent decrease in cell viability and increase in cell death (Figure 2G and H). Knockdown of *ALPL* was also confirmed in PC3 prostate cancer cells, and associated with a reduction in cell viability and an increase in cell death (Figure 2I–K).

### Inhibition of alkaline phosphatase activity induces MET and decreases migration in prostate cancer cells

**Figure 3:**
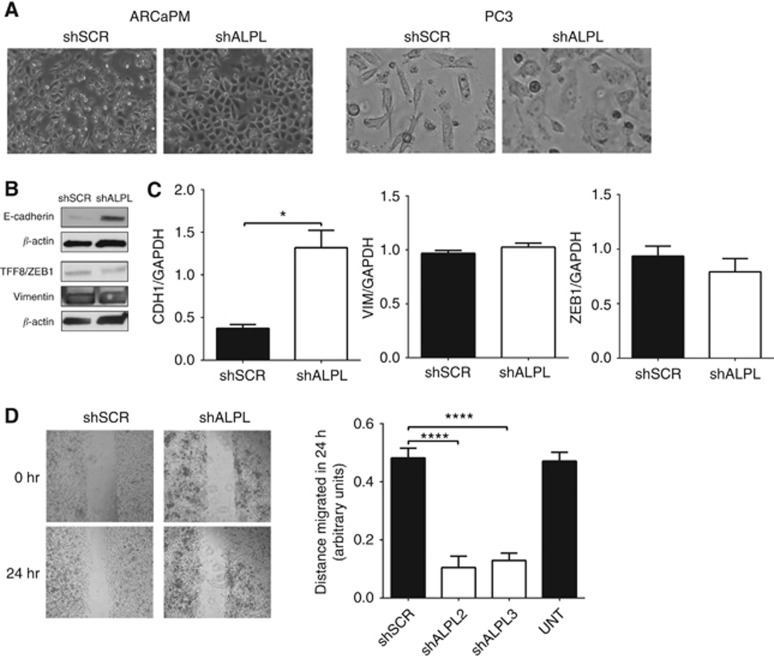
**Inhibition of alkaline phosphatase activity induces MET and decreases migration.** Prostate cancer cells transduced with scrambled shRNA (shSCR) or *ALPL*-knockdown shRNA (shALPL) were studied for features of EMT. (**A**) Cell morphology was documented by phase-contrast microscopy in ARCaPM cells and PC3 cells. (**B**) Protein levels and (**C**) mRNA expression of EMT markers were measured in ARCaPM cells using western blotting and qRT–PCR, respectively. (**D**) Cell migration was measured in ARCaPM using a scratch assay with light microscopy images taken at the indicated hours and quantitated using ImageJ. (*P<0.05, ****P<0.0001).

As our data suggested that alkaline phosphatase activity was increased in metastatic prostate cancer cells, we investigated the role of alkaline phosphatase in key metastatic features. ARCaPM cells exhibit a mesenchymal phenotype and *ALPL* knockdown resulted in a marked change in the morphology of ARCaPM cells from a spindle-shaped, scattered appearance to a rounded shape, with cells aggregating in clusters (Figure 3A). Similar to ARCaPM, knockdown of *ALPL* expression in PC3 prostate cancer cells was also associated with a change in morphology (Figure 3A). Associated with these morphological changes was a significant increase in the expression of the epithelial marker E-cadherin mRNA and protein (Figure 3B and C). Although no significant difference was detected in mRNA expression of the mesenchymal markers vimentin or ZEB1, a reduction in protein expression was observed (Figure 3B and C). Taken together, these suggest that knockdown of *ALPL* induces MET. In addition to MET, knockdown of *ALPL* resulted in a significant reduction in migration by up to 80% (Figure 3D).

### The EMT transcription factor Snail regulates alkaline phosphatase activity in prostate cancer cells

**Figure 4:**
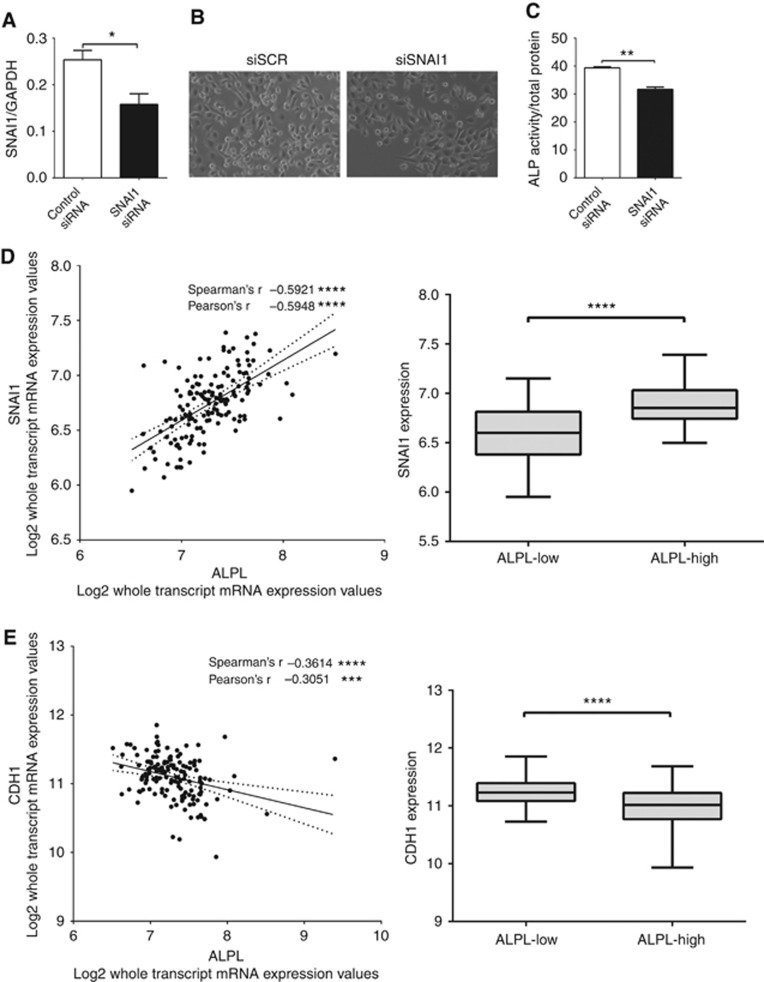
**Snail regulates expression of alkaline phosphatase *in vitro* and *in vivo*.** *In vitro*, Snail expression was inhibited by transfecting siRNA in ARCaPM cells and (**A**) knockdown was confirmed using qRT–PCR. (**B**) Change in morphology after Snail knockdown was documented using phase-contrast microscopy. (**C**) Alkaline phosphatase activity was measured in siRNA-transfected ARCaPM cells using a quantitative fluorimetric assay. The correlation of (**D**) Snail mRNA or (**E**) *CDH1* (E-cadherin) mRNA with *ALPL* mRNA expression was calculated in prostate cancer samples from the Taylor *et al.* data set (Taylor *et al*, 2010). In each case, the samples were also stratified into *ALPL*-high or *ALPL*-low samples (with median *ALPL* expression as the cutoff) and Snail or E-cadherin expression were plotted, respectively (^NS^*P*>0.05, *P<0.05, **P<0.01, ***P<0.001, ****P<0.0001).

Our results identify alkaline phosphatase as a novel regulator of EMT in prostate cancer cells, with high expression associated with metastatic disease; however, the effect of EMT on alkaline phosphatase remains unknown. To address this, expression of the key EMT transcription factor Snail was decreased in ARCaPM cells using siRNA. A significant reduction in Snail expression was confirmed by real-time PCR (Figure 4A), which induced a change from mesenchymal-to-epithelial morphology (Figure 4B). The decrease in Snail expression and resultant morphology change was associated with a significant decrease in alkaline phosphatase enzymatic activity (Figure 4C).

To determine whether alkaline phosphatase may regulate EMT in patients with prostate cancer, gene expression data from publicly available data sets were analysed. A significant positive correlation between *ALPL* expression and Snail expression was detected, concomitant with a significant negative correlation between *ALPL* expression and E-cadherin (Figure 4D). This is supported by a negative correlation between *ALPL* and E-cadherin gene expression in prostate cancer cells transduced with shALPL (Supplementary Data Figure S6). When gene expression data from patients with prostate cancer was stratified according to *ALPL* expression, Snail expression was significantly higher in *ALPL*-high samples, whereas E-cadherin expression was significantly lower (Figure 4E). To confirm the association between *ALPL* expression and EMT, two further independent data sets were analysed, with a significant positive correlation between *ALPL* expression and Snail expression detected in each study (Supplementary Data Figure S7) (Tomlins *et al*, 2007; Robinson *et al*, 2015).

### ATP induces MET and decreases viability, but does not mediate the effects of alkaline phosphatase

**Figure 5:**
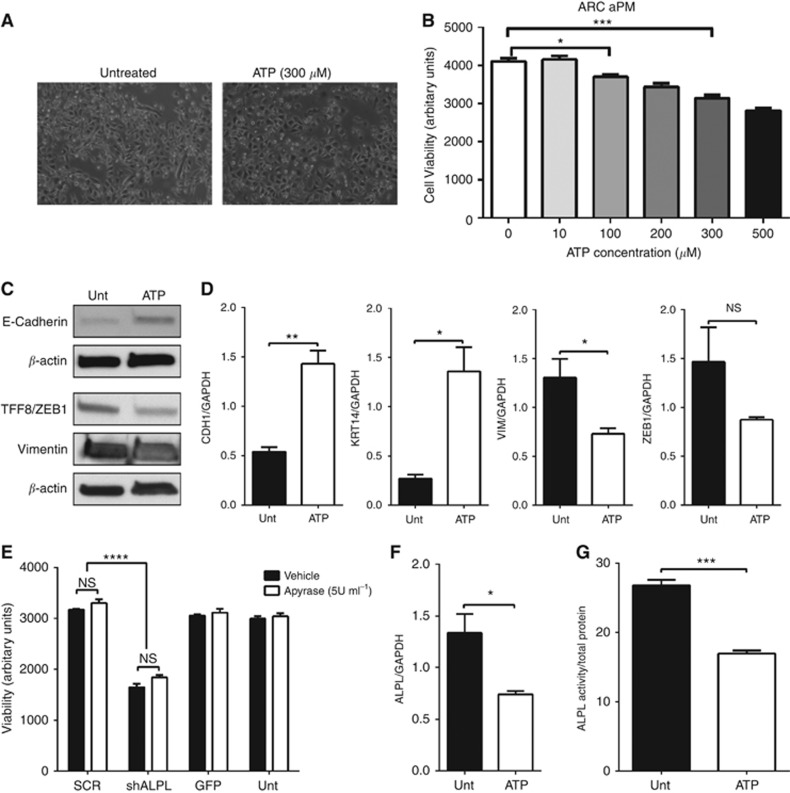
**ATP induces MET and decreases viability, but does not mediate the effects of tumour-derived alkaline phosphatase.** (**A**) ARCaPM cells were treated with exogenous ATP (300 *μ*M) for 24 h and cell morphology was documented using phase-contrast microscopy. (**B**) Viability was measured using Alamar Blue assay in ARCaPM cells treated with increasing concentrations of ATP. (**C**) Protein levels and (**D**) mRNA expression of EMT markers were measured in ARCaPM cells treated with ATP (300 *μ*M) for 24 h, using western blotting and qRT–PCR, respectively. (**E**) ARCaPM cells transduced with the indicated shRNA lentivirus were treated with vehicle or apyrase, and viability was measured using Alamar Blue assay. ALPL mRNA (**F**) and ALP activity (**G**) were measured in ARCaPM cells treated with ATP (300 *μ*M) for 24 h, using qRT–PCR and a quantitative fluorimetric assay, respectively. (^NS^*P*>0.05, *P<0.05, **P<0.01, ***P<0.001, ****P<0.0001).

One of the possible substrates of alkaline phosphatase is the nucleotide ATP, which is known to have direct antitumour effects in prostate cancer. Treatment of ARCaPM prostate cancer cells with 300 *μ*M ATP resulted in a change from an elongated spindle shape to a more rounded, epithelial-like morphology (Figure 5A) and a dose-dependent decrease in cell viability (Figure 5B). Further evidence for ATP induction of MET was provided by an increase in expression of epithelial markers E-cadherin and cytokeratin 14, and a reduction in mesenchymal markers vimentin and ZEB-1 (Figure 5C and D).

To determine whether ATP may mediate the effects of alkaline phosphatase, expression of *ALPL* was silenced in prostate cancer cells in the presence and absence of apyrase, which acts to degrade ATP (Supplementary Data Figure S8). The addition of 5 U ml^−1^ apyrase had no effect on the reduction in cell viability induced by a loss of *ALPL* expression, suggesting that degradation of ATP was not responsible for the effects of ATP in prostate cancer cells (Figure 5E). In contrast, treatment of prostate cancer cells with ATP was found to significantly reduce alkaline phosphatase gene expression and activity (Figure 5F and G).

### High expression of tumour-derived alkaline phosphatase is associated with decreased disease-free survival in prostate cancer patients

**Figure 6:**
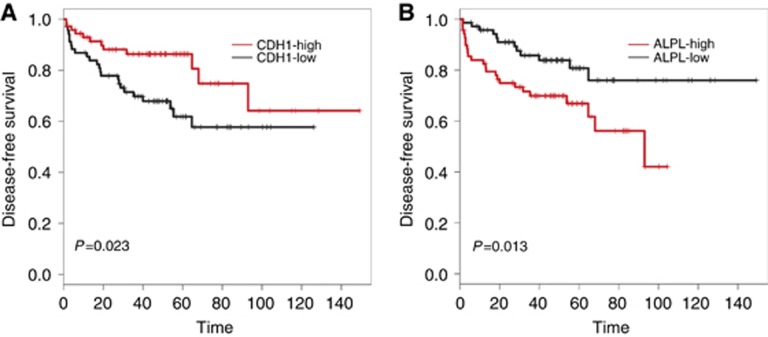
**High tumour alkaline phosphatase gene expression is associated with decreased disease-free survival in prostate cancer patients.** Prostate cancer samples (*n*=150) from the Taylor *et al.* data set (Taylor *et al*, 2010) were stratified as high and low, with median gene expression (*CDH1* or *ALPL*, as indicated) as the cutoff. Disease-free survival was plotted for (**A**) *CDH1*-high *vs CDH1*-low and (**B**) *ALPL*-high *vs ALPL*-low samples (*P*-value is obtained using log-rank test).

Our studies have identified tumour-derived alkaline phosphatase as a novel mediator of prostate cancer cell growth and EMT. To determine the clinical significance of alkaline phosphatase expression, we analysed gene expression as associated with disease-free survival in men with prostate cancer. Samples from the Taylor *et al.* data set (Taylor *et al*, 2010) were stratified into ‘high' or ‘low' based on *ALPL* or *CDH1* (E-cadherin) expression, with the median expression as the cutoff. High expression of E-cadherin was associated with an increase in disease-free survival (Figure 6A). In contrast, high expression of *ALPL* in prostate cancer cells was associated with a significant reduction in disease-free survival (Figure 6B and Table 1).

## Discussion

Alkaline phosphatase activity in serum, a measure of alkaline phosphatase expressed by osteoblasts, has long been used as an indicator of increased bone remodelling, and hence, bone metastatic disease in prostate cancer (Brown and Sim, 2010). Activity is thought to reflect alkaline phosphatase originating from bone, liver and kidney. Our data demonstrate that alkaline phosphatase is expressed in prostate cancer cells, with a significant differential expression in mesenchymal prostate cancer cells compared with their epithelial counterpart. This is supported by our analysis of the Tomlins *et al.* data set (Tomlins *et al*, 2007), where expression of alkaline phosphatase in metastatic samples was much higher compared with primary prostate cancer samples. Importantly, this data set was generated from prostate cancer cells isolated by laser capture microdissection from the primary or metastatic site, thus removing the risk of contamination from surrounding stromal cells. The clinical importance of tumour-derived alkaline phosphatase in prostate cancer is evident from the significant decrease in disease-free survival in patients with high expression of alkaline phosphatase in the Taylor *et al.* data set (Taylor *et al*, 2010).

Alkaline phosphatase is a defining marker of osteoblast activity (Wennberg *et al*, 2000), and as such alkaline phosphatase expression by prostate cancer cells provides further support for the concept of osteomimicry, the process by which tumour cells express bone-specific markers, which may allow them to survive better in the bone microenvironment during metastatic colonisation (Koeneman *et al*, 1999; Rucci and Teti, 2010). In the present study, alkaline phosphatase expression in the panel of prostate cancer cell lines was not directly associated with tumourigenicity or with the bone-destructive nature of the cell lines, suggesting that tumour-derived alkaline phosphatase does not merely promote osteoblastic bone disease. Instead, reduced alkaline phosphatase expression or activity in prostate cancer cells was found to induce MET and prostate cancer cell death, revealing a novel role for this gene in tumour cell biology. While the most striking phenotype of knocking out alkaline phosphatase in mice is neurological abnormalities (characterised by early-onset seizures) and diminished bone mineralisation, there is evidence of increased apoptosis in the thymus (Waymire *et al*, 1995; Fedde *et al*, 1999). Knockdown of alkaline phosphatase in murine osteoblasts also results in increased apoptosis (Liu *et al*, 2014). These studies support our findings whereby loss of alkaline phosphatase expression or activity results in an increase in prostate cancer cell death. The high concentrations of the alkaline phosphatase inhibitor levamisole required to induce cell death suggest that either these effects of levamisole are independent of alkaline phosphatase inhibition or that a stronger inhibition of alkaline phosphatase activity is required to induce prostate cancer cell death, as compared with reduce osteoblast mineralisation. As EMT is associated with resistance to apoptosis (Robson *et al*, 2006), it is intriguing to speculate that the increase in cell death is a result of the concomitant MET. In addition to an increase in cell death, loss of alkaline phosphatase was associated with MET and a reduction in migration. Knockdown of alkaline phosphatase in ARCaPM cells resulted in an increase in E-cadherin mRNA expression, but there was no change in the mRNA expression of the mesenchymal markers vimentin and ZEB1, suggesting that the MET observed could be partial (Leroy and Mostov, 2007). However, there was a clear decrease in the protein levels of vimentin and ZEB1, raising the possibility of a post-transcriptional regulatory mechanism. Knockdown of the key EMT transcription factor Snail resulted in reduced alkaline phosphatase activity, suggesting a mechanism by which alkaline phosphatase may be regulated. In human samples from the Taylor *et al.* data set Taylor *et al*, 2010, alkaline phosphatase expression was positively correlated with Snail expression and negatively correlated with E-cadherin expression, providing clinical evidence to support our *in vitro* findings.

The transformation of epithelial cells to a motile mesenchymal phenotype has long been considered as a key step in the metastatic cascade, allowing tumour cells to invade their surrounding stroma and ultimately migrate to their metastatic site. Within the bone marrow microenvironment, there is evidence to suggest that cells can revert back to their epithelial phenotype, although the frequency and function of this MET is poorly understood (van der Pluijm, 2011). The current study identifies tumour-derived alkaline phosphatase as an important regulator of epithelial plasticity, associated with metastasis and disease-free survival in patients with prostate cancer. Alkaline phosphatase is well characterised as a marker of pluripotent stem cells, and as EMT has been associated with the acquisition of cancer stem cell properties, it is intriguing to speculate that alkaline phosphatase expression may contribute to the development of a cancer stem cell state (Stefkova *et al*, 2015; Ye and Weinberg, 2015).

One of the major substrates of alkaline phosphatase is ATP, and Díez-Zaera *et al* (2011) demonstrated that alkaline phosphatase regulates the growth of neuronal axons by affecting the levels of extracellular ATP. As extracellular ATP was previously shown to be an inducer of cell death in prostate cancer (Shabbir *et al*, 2008), we hypothesised that the effects of alkaline phosphatase knockdown in prostate cancer cells may be mediated by the increased levels of extracellular ATP, with differing concentrations of extracellular ATP well known to activate distinct purinergic receptors such as the P2X7 receptor. However, despite the similarity of their effects in inducing MET, our data with apyrase treatment suggest that extracellular ATP does not have a role in alkaline phosphatase knockdown-induced reduction in cell viability. Hence, the exact mechanism by which alkaline phosphatase knockdown causes MET and cell death in prostate cancer cells remains to be explored. It is interesting to note that ATP treatment induced a reduction in alkaline phosphatase mRNA expression and activity in prostate cancer cells, similar to the effects seen in osteoblasts (Orriss *et al*, 2007).

Our studies identify tumour-derived alkaline phosphatase as elevated in metastatic prostate cancer, associated with reduced disease-free survival. These findings, when combined with the elevated circulating alkaline phosphatase levels that are associated with advanced prostate cancer, identify alkaline phosphatase as a potential therapeutic target. In addition to the association with disease-free survival identified in the present study, increased expression of alkaline phosphatase is associated with vascular calcification. Considerable advances have been made to identify small-molecule inhibitors of TNAP for the treatment of diseases associated with arterial medial calcification (Narisawa *et al*, 2007; Sidique *et al*, 2009), and our results suggest that these inhibitors would be of interest to evaluate in the setting of metastatic prostate cancer. Recent studies have demonstrated that bone-targeted alkaline phosphatase can act as enzyme-replacement therapy for hypophosphatasia, highlighting the promise of alkaline phosphatase as a therapeutic target (Whyte *et al*, 2012).

The inevitable fatality associated with advanced prostate cancer and bone metastasis, and the unpredictable nature of progression from primary prostate cancer make it imperative to identify mechanisms that will ultimately enhance our understanding of disease progression, leading to new therapeutic approaches or prognostic indicators. Tumour-derived alkaline phosphatase represents one such mechanism, where a previously unrecognised role in tumour cell biology is associated with disease progression. Collectively, our studies have identified a novel role for tumour-derived alkaline phosphatase in MET and cell death. Moreover, we have highlighted the clinical association between tumour-derived alkaline phosphatase expression and metastatic disease, further supported by a significant decrease in disease-free survival in prostate cancer patients with high alkaline phosphatase expression.

## Figures and Tables

**Table 1:** Survival analysis in ALPL-low and ALPL-low samples (MSKCC data set)

		**Relapsed cases**		
**ALPL status**	**Total cases with survival data (total cases)**	**Observed**	**Expected**	**Median months disease free**
Low	71 (75)	12	19.4	NA
High	69 (75)	24	16.6	92.98

**Hazard ratio (log rank)**		
	**ALPL-low**	**ALPL-high**
Ratio (and its reciprocal)	0.4261	2.347
95% CI of ratio	0.2254–0.8360	1.196–4.437

Abbreviations: CI=confidence interval; d.f.=degrees of freedom; MSKCC=Memorial Sloan Kettering Cancer Center; NA=not applicable.

*χ*^2^=6.2 on 1 d.f., *P*=0.0127; *n*=140, and 10 observations were deleted owing to missingness.
