# Supplementary material for: Tumour-derived alkaline phosphatase regulates tumour growth, epithelial plasticity and disease-free survival in metastatic prostate cancer
Source: Br J Cancer. 2016 Dec 22;116(2):227–36. doi: 10.1038/bjc.2016.402 (PMC5243990; doi:10.1038/bjc.2016.402)
Supplement: Supplementary Information [file bjc2016402x1.pdf]

Supplemental Data

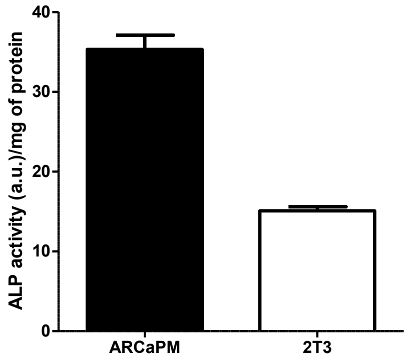

Figure S1. Alkaline phosphatase activity in prostate cancer cells and osteoblasts. Alkaline phosphatase activity was measured in ArCAPM prostate cancer cells and 2T3 osteoblasts, and normalised to mg protein.

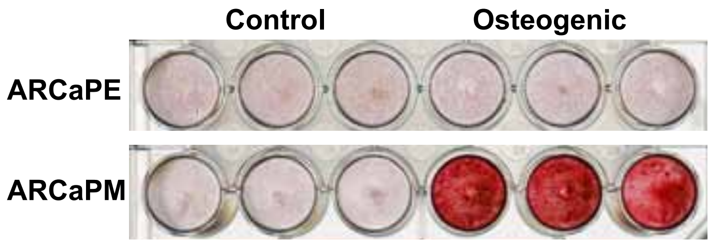

Figure S2. ARCaPM, not ARCaPE prostate cancer cells mineralise in vitro. ARCaPE and ARCaPM cells were grown in control or osteogenic medium for 21 days and stained (red) for mineral deposition using Alizarin Red S.

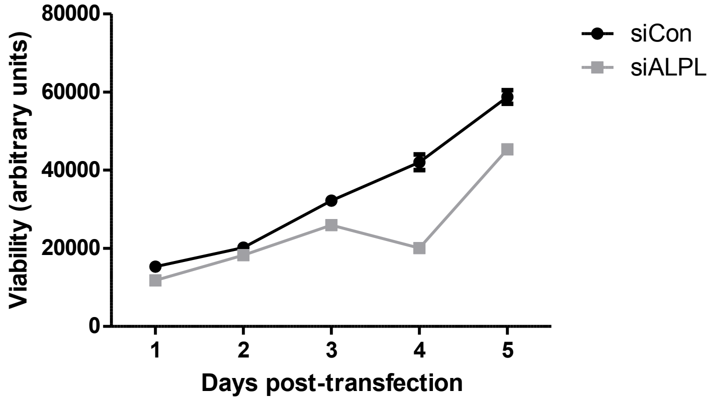

Figure S3. Inhibition of alkaline phosphatase decreases prostate cancer cell viability. ArCAPM prostate cancer cells were transfected with siALPL or scrambled control, and cell viability measured at 24h intervals following transfection.

Supplemental Data

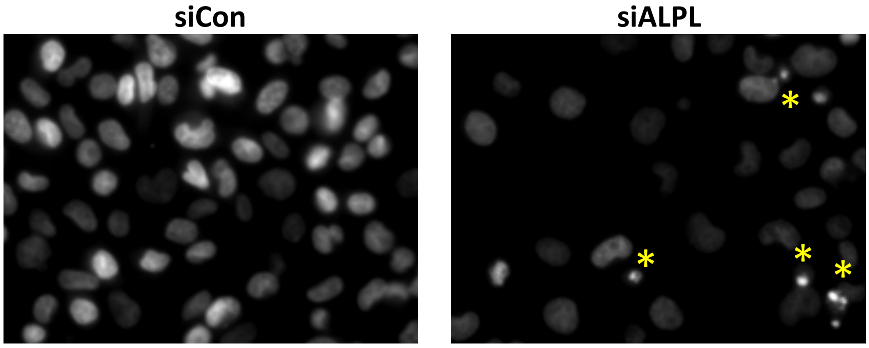

Figure S4. Inhibition of alkaline phosphatase induces prostate cancer cell apoptosis. *ALPL* was knocked down in ARCaPM cells following transduction with scrambled control (siCon) or ALPL-siRNA (siALPL). Cells were fixed and stained with DAPI, and nuclear morphology visualised by microscopy. Apoptotic nuclei are indicated by *.

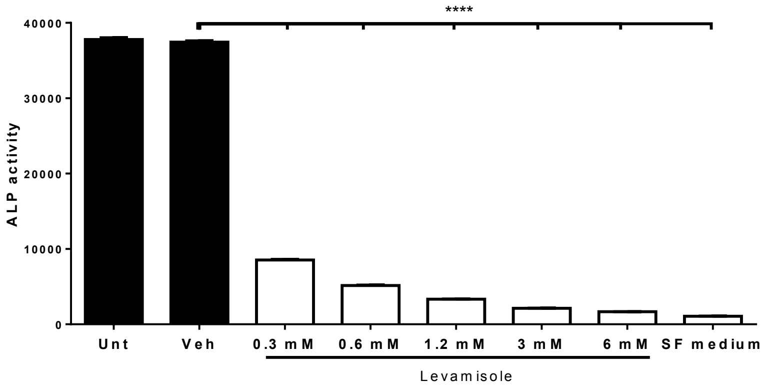

Figure S5: Levamisole inhibits alkaline phosphatase activity in growth medium. Levamisole was added to growth medium with 10% FBS at the indicated concentrations and alkaline phosphatase enzyme activity was measured following incubation at 37°C for 1 hour. (SF medium: serum-free medium, without addition of levamisole. **** P < 0.0001).

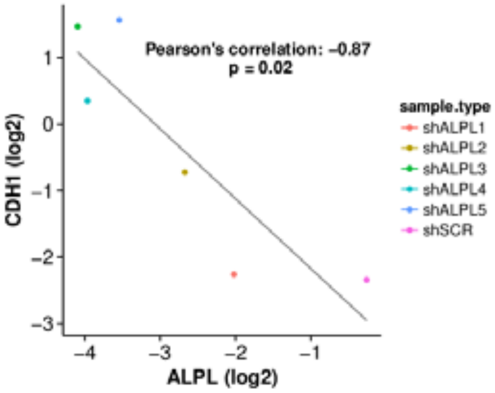

Figure S6. Negative correlation between *ALPL* and E-cadherin (CDH1) mRNA expression in ARCaPM cells. mRNA expression of ALPL and CDH1 was measured, using qRT-PCR (normalized to GAPDH expression), in ARCaPM cells transduced with 5 different ALPL-targeting shRNA constructs (shALPL1-5) or a scrambled control (shSCR).

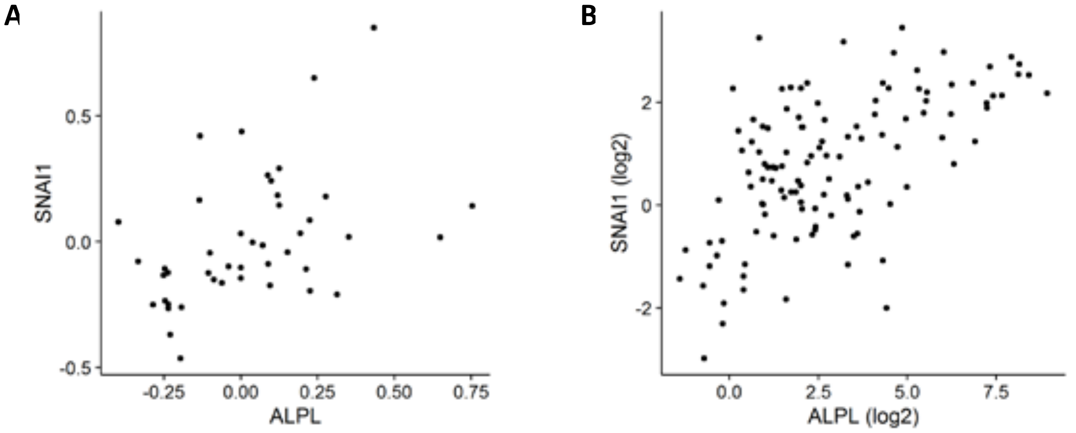

Figure S7. Correlation between *ALPL* and *SNAIL* mRNA expression. mRNA expression of *ALPL* and *Snail* were fetched from the (A) Tomlins et al. (r = 0.43, p < 0.01) and (B) Robinson et al. (r = 0.57, p < 0.001) datasets.

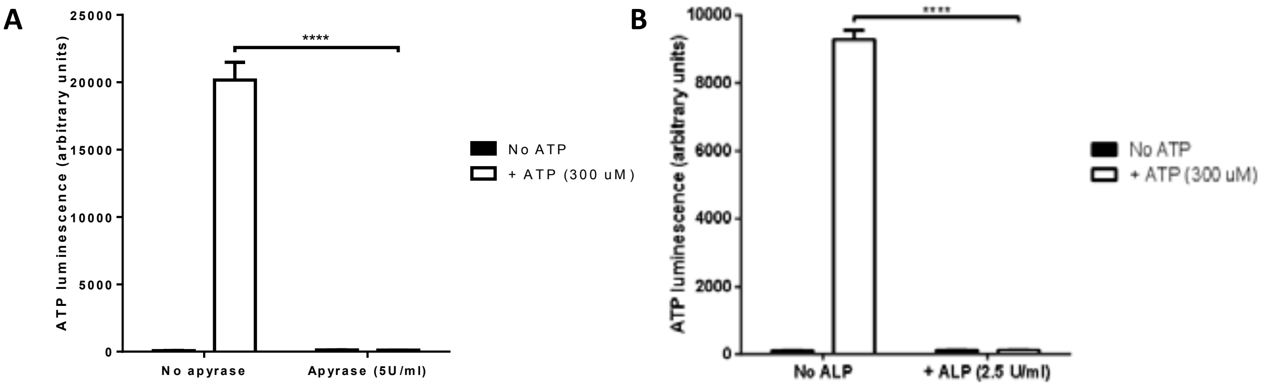

Figure S8. ATP is a substrate for apyrase and alkaline phosphatase. ATP was added to serum-free growth medium and incubated with (A) apyrase or (B) alkaline phosphatase, at 37^{0}C for 1 hour and ATP levels were subsequently measured with an ATP assay kit. (**** P < 0.0001)
